# Effects of Water Quality on Dissolution of Yerba Mate Extract Powders

**DOI:** 10.1155/2014/768742

**Published:** 2014-02-24

**Authors:** Wen-Ying Huang, Pei-Chi Lee, Jaw-Cherng Hsu, Yu-Ru Lin, Hui-Ju Chen, Yung-Sheng Lin

**Affiliations:** ^1^Department of Applied Cosmetology and Master Program of Cosmetic Science, Hungkuang University, Taiwan Boulevard, Shalu District, Taichung 43302, Taiwan; ^2^Department of Cosmetic Science, National Tainan Junior College of Nursing, West Central, Tainan 70043, Taiwan

## Abstract

Yerba mate tea is known as one of the most popular nonalcoholic beverages favoured by South Americans due to its nutrition facts and medicinal properties. The processing of yerba mate tea is found to affect the properties of its final forms. This study presents an investigation into the effects of water sources on the dissolution of yerba mate extract powders. Comparisons were conducted between yerba mate teas prepared by dissolving yerba mate extract powders into tap water and deionized water. Topics to be explored in this work are the major compositions and antioxidant activities, including total phenol content, reducing power, DPPH scavenging activity, and ABTS^+^• scavenging capacity. It is indicated that there is little difference for antioxidant activities and major constituents of yerba mate teas between both water sources. However, a deeper color is seen in the tap water case, resulting from the reaction between tannic acid and ions. This research finding can be treated as a way to benefit the yerba mate tea processing for applications.

## 1. Introduction 

Yerba mate (*Ilex paraguariensis*), a traditional crop, is known as one of the most popular beverages in Argentina, Brazil, Paraguay, and Uruguay [1]. As a nonalcoholic beverage favoured by South Americans [2], yerba mate provides a great number of bioactive compounds for nutritional and medicinal applications. On account of its alleged therapeutic capacity, yerba mate is found to have hypocholesterolemic, hepatoprotective, and diuretic properties and can resist against the deleterious effect of free radicals, thus boosting the defense system of organisms [3–8]. Furthermore, it can as well improve the cardiovascular system in a human body [9–11].

Polyphenols (chlorogenic acid) and xanthines (caffeine and theobromine) rank the top 2 places in the sorting by the level of active phytochemicals contained in yerba mate tea [8, 12]. The antioxidant activity of the phenolic compounds is mainly due to the redox properties thereof, which enable them to serve as good reducing agents [13]. The considerable antioxidant potential of yerba mate has long been recognized and shows dependence on many factors involved in tea preparation.

Various processing procedures of plant leaves will result in different tea qualities [14–18]. There are a number of published studies on the effect of aqueous infusion on yerba mate tea. Isolabella et al. [1] and Valerga et al. [19] made an investigation into compound variation during each step of its industrial process. Linares et al. [20] studied the temperature influence on the aqueous infusion of yerba mate. In contrast, ion effects on aqueous infusion of yerba mate have rarely been described. According to previous reports on green tea [21, 22], elements of water are presumed to have some influence on yerba mate tea.

In consideration of the demand growth and potential health benefits from drinking yerba mate tea, this study is proposed to quantize the ion effects on the dissolution of yerba mate extract powders. Comparisons are conducted on the contents of major bioactive components and the antioxidant activities of yerba mate tea prepared by two water sources. Results of this study shed light on the effect of yerba mate tea preparation toward a better understanding of the tea quality in its final presentation.

## 2. Materials and Methods

### 2.1. Chemicals and Reagents

Panted Finomate EFLA 920 yerba mate extract was purchased from Frutarom Switzerland Ltd., Wädenswil, Switzerland. Folin-Ciocalteu reagent was from Fluka (Neu-Ulm, Germany). Iron(III) chloride was from Riedel-de Haen (Seelze, Germany). Tannic acid, caffeine, sodium carbonate, trichloroacetic acid (TCA), 2,2-diphenyl-1-picrylhydrazyl (DPPH), butylated hydroxyanisole (BHA), and 2,2′-azino-bis(3-ethylbenzothiazoline-6-sulphonic acid) (ABTS) were from Sigma (St. Louis, USA). Potassium persulfate was from Showa Chemical Co. (Tokyo, Japan). Potassium ferricyanide (K_3_Fe(CN)_6_) was from JT Baker (Phillipsburg, USA). Potassium dihydrogen phosphate (HPLC grade) and phosphoric acid (HPLC grade) were from Wako Pure Chemical Industries, Ltd. (Tokyo, Japan). Methanol and acetonitrile (HPLC grade) were from Merck (Darmstadt, Germany).

### 2.2. Processing of Yerba Mate Tea

Yerba mate tea solutions were prepared at 25°C by dissolving yerba mate extract powders into tap water and deionized water, respectively. The former was taken from the suburban area of the Greater Taichung area, Taiwan. Deionized water was purified with a Milli-Q water system (Millipore, Bedford, USA). Yerba mate tea was prepared by dissolving different amounts of dry yerba mate extract powders in 25 mL water. Three replicates were made in each experiment for statistical analysis.

### 2.3. Total Phenol Content

Total phenol content of the yerba mate tea was estimated by a colorimetric assay based on the procedure described by Kumazawa et al. [23]. The 0.1 mL of yerba mate tea solution (100~500 ppm) was mixed with 0.5 mL of Folin-Ciocalteu reagent for a duration of 1 minute and 0.5 mL of 2% sodium carbonate for 20 minutes. The absorbance at 655 nm (Sunrise ELISA plate reader, Tecan, Austria) increases with the total phenol content.

### 2.4. Reducing Power

The reducing power of yerba mate tea was examined using a mixture of 1 mL of phosphate buffer (0.2 M, pH 6.6), 1 mL of potassium ferricyanide (1% by weight), and 1 mL of tested sample [24]. The mixture was incubated at 50°C for 20 minutes, and a 1 mL volume of TCA (1% by weight) was then added to the mixture. The TCA-reacted solution (0.4 mL) was mixed with deionized water (0.5 mL) and FeCl_3_ (1 mL, 0.1% by weight) for 10 minutes, and the absorbance was measured at 700 nm.

Relative reducing power (%) is defined as relative reducing power = (*A*/*B*) × 100, where *A* denotes the sample absorbance and *B* the absorbance of 0.5 mg/mL BHA.

### 2.5. DPPH Scavenging Activity

The DPPH scavenging ability assay was prepared as in Zhang and Wang [25]. A 0.1 mL of sample was mixed with 1.0 mL of 0.25 mM DPPH and 0.4 mL of ethanol, incubated for 30 minutes in the dark. When DPPH reacted with an antioxidant that can donate hydrogen, it appeared in reduced form and resulted in an absorbance drop at 517 nm (Sunrise ELISA plate reader, Tecan, Austria). The DPPH scavenging activity is defined as follows:

(1) DPPH  scavenging  activity  (%) =(1−A517  of  sampleA517  of  control)×100,

where the control denotes the water not containing yerba mate tea.

### 2.6. ABTS^+^•**  **Scavenging Capacity

The ABTS^+^•**  **scavenging capacity assay was carried out using the procedure described in Erkan et al.'s method [26]. In brief, ABTS^+^•**  **was generated by the reaction of 7 mM of ABTS with 2.45 mM of potassium persulfate in the dark for 16 hours at 4°C. The 10 *μ*L of yerba mate tea sample was added to 2 mL of ABTS^+^•**  **radical solution, for a reaction time of 10 minutes. Given the absorbance at 734 nm, the ABTS^+^•**  **radical scavenging activity is defined as

(2) ABTS+•  radical  scavenging  (%) =(1−A734  of  sampleA734  of  control)×100,

where the control represents the water not containing yerba mate tea.

### 2.7. High Performance Liquid Chromatography

Each extracted yerba mate tea sample was mixed (1 : 1, v/v) with a gallic acid solution (internal standard, 20 mg/L in 70% methanol). Subsequently, all the samples were spiked with various concentrations of stock solutions (theobromine: 3.125, 3.57, 4.17, 8.33, and 12.5 *μ*g/mL; chlorogenic acid: 7.143, 8.33, 10, 16.67, and 25 *μ*g/mL; caffeine: 5, 6.25, 8.33, 10, and 15.625 *μ*g/mL) in advance of the HPLC analysis. A 20 *μ*L of these solutions was injected twice by the HPLC method, respectively, and the standard curves were plotted according to the peak areas versus concentrations. Recovery was determined by the comparison between the amounts of marker-substances added and found. The detection limits arise from a minimum signal to noise (*S*/*N*) ratio of 3. The HPLC system (Agilent 1200 infinity series, Agilent, USA) is equipped with a quaternary pump, an autosampler, a vacuum degasser, and a diode array detector. A reverse phase column (Cosmosil 5C18-AR II, 5 *μ*m, 25 cm × 4.6 mm I.D., Nacalai Tesque, Kyoto, Japan) was used. The mobile phase was a mixture of A (10 mM KH_2_PO_4_, pH 4.0) and B [CH_3_CN : CH_3_OH : H_2_O = 1.5 : 2.5 : 1 (v/v/v)]. The initial mobile phase composition was 20% of B. After 20 min, the mobile phase composition turned into 30% of B. After 40 min, the mobile phase composition further turned into 50% of B. The flow rate was 0.8 mL/min. The detector was operated at a detection wavelength of 280 nm.

## 3. Results and Discussion

### 3.1. Solution Appearance

**Figure 1:**
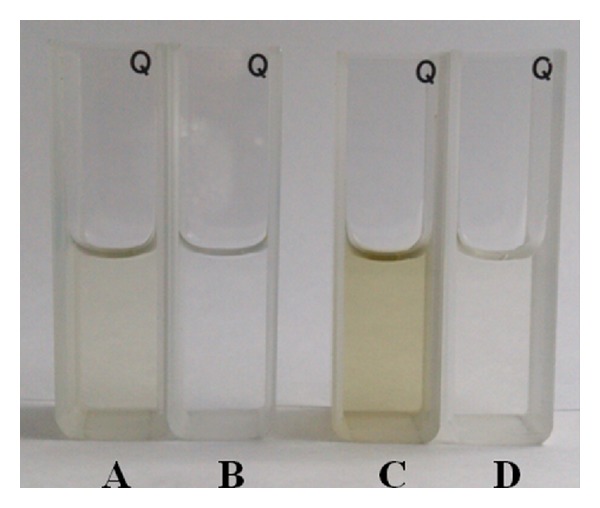
Color appearance comparison between yerba mate tea solutions. A and C are prepared with tap water and B and D are with deionized water. A and B have a tea concentration of 100 ppm while C and D have 500 ppm.

Figure 1 indicates the appearance of yerba mate tea solution placed in quartz cuvettes. As such, the color darkens as the concentration of yerba mate tea rises. A concentrated solution (500 ppm, C and D in Figure 1) demonstrates a deeper color than a diluted one (100 ppm, A and B in Figure 1). At the same level of tea concentration, yerba mate tea prepared with tap water shows a darker orange color than prepared with deionized water. The color difference is presumed to reflect ion chelation with the composition of yerba mate tea.

**Figure 2:**
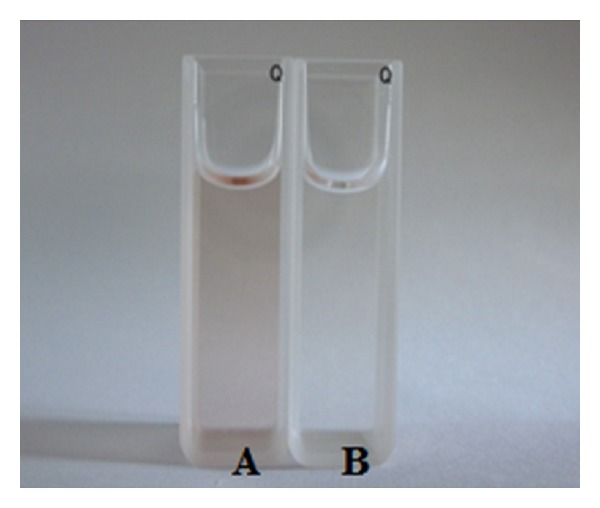
Color comparison between tannic acid solutions at a 250 ppm concentration prepared with tap water (A) and deionized water (B).

For a further investigation into the color difference between yerba mate teas prepared with different water sources, the issue of dissolution of tannic acid, a compound contained in yerba mate tea is addressed.  Figure 2 shows the color comparison between the tannic acid solutions prepared with tap water and deionized water. There is a deeper color in the tap water than in the deionized water case, an event that may be attributed to the ion chelating between tannic acid and metal ions in tap water.

### 3.2. Total Phenol Content

**Figure 3:**
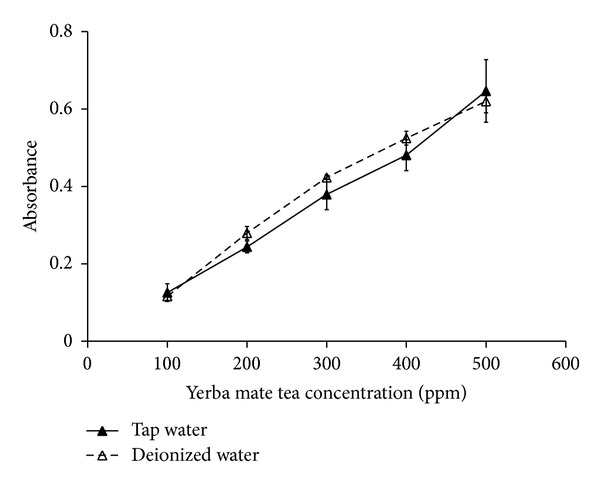
Comparison of the total phenol content versus the concentration of yerba mate tea.

Phenols are regarded as the significant constituents of yerba mate tea, and the concentration thereof is directly proportional to the absorbance at 655 nm (*A*_655_) in Folin-Ciocalteu reaction.  Figure 3 is a plot of *A*_655_ versus the yerba mate tea concentration, where the yerba mate tea concentration is directly reflected by the total phenol contents. Yet, there is little difference in *A*_635_ between the tap water and the deionized water cases, meaning that the total phenol content is marginally affected by the choice of water sources.

### 3.3. Reducing Power

**Figure 4:**
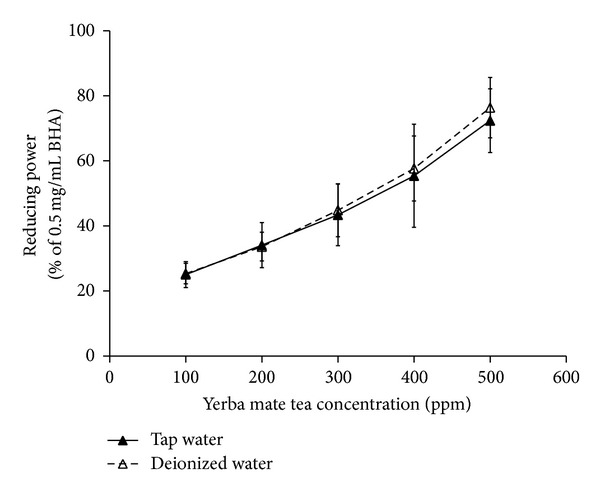
Comparison of the reducing power versus the concentration of yerba mate tea.

The reducing power of a compound is seen as a measure of antioxidant activity.  Figure 4 exhibits the reducing power comparison between both test cases. A 0.5 mg/mL BHA solution was used as a reference in the reducing power measurement. As it turns out, there is a nearly linear relationship between the reducing power and tea concentration, even at a tea concentration of 500 ppm (72.4 ± 9.8% versus 76.38 ± 9.27%), and a good agreement between both samples.

### 3.4. DPPH Scavenging Activity

**Figure 5:**
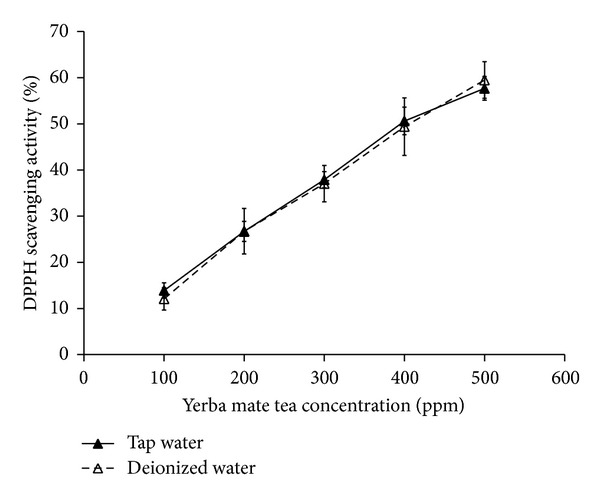
Comparison of the DPPH scavenging activity versus the concentration of yerba mate tea.

As a stable free radical compound, DPPH has been widely adopted as a measure of free radical scavenging activity. This reduction can be monitored at 517 nm by measuring the bleaching of a violet DPPH solution.  Figure 5 shows the DPPH radical scavenging activity of yerba mate tea. There is a dose-dependent relationship between the DPPH scavenging activity and the concentration of yerba mate tea and a high consistency between both test samples over the while observation. Respective 50% DPPH radical scavenging activities (IC_50_) are observed at tea concentrations of 413 and 411 ppm in the tap water and the deionized water cases. Hence, the DPPH scavenging activity shows no dependence on the ion concentrations of water.

### 3.5. ABTS^+^•**  **Scavenging Activity

**Figure 6:**
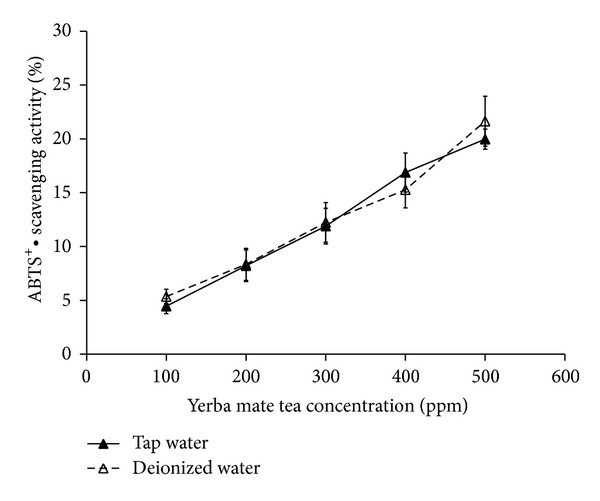
Comparison of the ABTS^+^•**  **scavenging activity versus the concentration of yerba mate tea.

ABTS^+^•**  **scavenging activity is another indicator used to test antioxidant activity of yerba mate tea.  Figure 6 reveals a dose-dependent ABTS^+^•**  **radicals scavenging activity for yerba mate tea. ABTS^+^•**  **radicals were inhibited by 19.97 ± 0.94% and 21.64 ± 2.31% at a tea concentration of 500 ppm in the tap water and the deionized water cases, respectively. As the DPPH scavenging activity was discussed in the preceding section, results in Figure 6 demonstrate no dependence on the choice of water sources.

### 3.6. High Performance Liquid Chromatography

As a prerequisite for the establishment of calibration curves, the peak-area ratios are plotted against the tea concentrations. All the regression lines were linear over the concentration range of interest, that is, *y* = 57.9903*x* + 0.0296  (*R* ^2^ = 0.9983) for theobromine, *y* = 97.442*x* + 0.0525  (*R* ^2^ = 0.9998) for chlorogenic acid, and *y* = 120.15*x* + 0.0787  (*R* ^2^ = 0.9941) for caffeine. The respective detection limits (*S*/*N* = 3) for the components were 0.357 (theobromine), 0.714 (chorogenic acid), and 0.412 (caffeine) *μ*g/mL. Appropriate amounts of marker substances (theobromine, 6.25 *μ*g/mL, chorogenic acid, 12.5 *μ*g/mL, and caffeine, 12.5 *μ*g/mL) were added to a sample containing a known content and the mixtures were then analyzed by the proposed HPLC approach. The recoveries of the components are sorted in descending order, that is, 104.69, 99.09 and 91.06% for theobromine, chorogenic acid and caffeine, respectively.

Substituting the peak-area ratios of the individual peaks for *y* in the above-stated equations gives the contents of the individual components in the yerba mate tea. Tabulated in Table 1 was a comparison of the average amounts of the three aforementioned constituents contained in samples between both water cases. It is found that both cases contain comparable amounts of constituents.

Previous studies reported that mineralisation of water hindered tea leaves extraction of organic matter due to the complex formation [27, 28]. The work presents an investigation into the influence of water ions on the dissolution of yerba mate extract powders. Unlike the extractions of organic matters from tea leaves, the research findings indicate little differences in the compositions as well as the functional characteristics between yerba mate teas prepared by dissolving yerba mate extract powders into tap water and deionized water.

## 4. Conclusions

A difference in color appearance is seen between yerba mate teas prepared with tap water solution and deionized water. The deep color in a tap water case may be attributed to the ion chelating between tannic acid and metal ions contained in tap water. Analysis of high performance liquid chromatography and antioxidant experiments, including total phenol content, reducing power, DPPH scavenging activity, and ABTS^+^•**  **scavenging activity, reveals that yerba mate teas contain identical compositions and provide the same level of antioxidant activities irrespective of the choice of water sources. This finding can be useful for the processing of yerba mate.

## Figures and Tables

**Table 1:** Three major components contained in yerba mate teas prepared with distinct water solutions.

Water solution	Theobromine (mg/g)	Chlorogenic acid (mg/g)	Caffeine (mg/g)
Deionized water	45.873 ± 0.660	58.186 ± 0.542	43.865 ± 2.486
Tap water	48.403 ± 0.845	58.018 ± 1.161	45.180 ± 0.829

## References

[1] Isolabella S, Cogoi L, López P, Anesini C, Ferraro G, Filip R (2010). Study of the bioactive compounds variation during yerba mate (Ilex paraguariensis) processing. *Food Chemistry*.

[2] Small E, Catling PM (2001). Blossoming treasures of biodiversity: 3. Mate (Ilex paraguariensis)-better than Viagra, marijuana, and coffee?. *Biodiversity*.

[3] Filip R, Lotito SB, Ferraro G, Fraga CG (2000). Antioxidant activity of Ilex paraguariensis and related species. *Nutrition Research*.

[4] Filip R, López P, Giberti G, Coussio J, Ferraro G (2001). Phenolic compounds in seven South American Ilex species. *Fitoterapia*.

[5] Filip R, Ferraro GE (2003). Researching on new species of “Mate”: Ilex brevicuspis: phytochemical and pharmacology study. *European Journal of Nutrition*.

[6] Anesini C, Ferraro G, Filip R (2006). Peroxidase-like activity of Ilex paraguariensis. *Food Chemistry*.

[7] Colpo G, Trevisol F, Teixeira AM (2007). Ilex paraguariensis has antioxidant potential and attenuates haloperidol-induced orofacial dyskinesia and memory dysfunction in rats. *Neurotoxicity Research*.

[8] Martins F, Suzan AJ, Cerutti SM (2009). Consumption of mate tea (Ilex paraguariensis) decreases the oxidation of unsaturated fatty acids in mouse liver. *British Journal of Nutrition*.

[9] Schinella GR, Troiani G, Dávila V, De Buschiazzo PM, Tournier HA (2000). Antioxidant effects of an aqueous extract of Ilex paraguariensis. *Biochemical and Biophysical Research Communications*.

[10] Mosimann AL, Wilhelm-Filho D, Da Silva EL (2006). Aqueous extract of Ilex paraguariensis attenuates the progression of atherosclerosis in cholesterol-fed rabbits. *BioFactors*.

[11] Strassmann BB, Vieira AR, Pedrotti EL, Morais HNF, Dias PF, Maraschin M (2008). Quantitation of methylxanthinic alkaloids and phenolic compounds in mate (Ilex paraguariensis) and their effects on blood vessel formation in chick embryos. *Journal of Agricultural and Food Chemistry*.

[12] Heck CI, de Mejia EG (2007). Yerba mate tea (*Ilex paraguariensis*): a comprehensive review on chemistry, health implications, and technological considerations. *Journal of Food Science*.

[13] Atoui AK, Mansouri A, Boskou G, Kefalas P (2005). Tea and herbal infusions: their antioxidant activity and phenolic profile. *Food Chemistry*.

[14] Huang Y, Sheng J, Yang F, Hu Q (2007). Effect of enzyme inactivation by microwave and oven heating on preservation quality of green tea. *Journal of Food Engineering*.

[15] Sinija VR, Mishra HN, Bal S (2007). Process technology for production of soluble tea powder. *Journal of Food Engineering*.

[16] Komes D, Horžić D, Belščak A, Ganić KK, Vulić I (2010). Green tea preparation and its influence on the content of bioactive compounds. *Food Research International*.

[17] Jun X, Deji S, Ye L, Rui Z (2011). Comparison of in vitro antioxidant activities and bioactive components of green tea extracts by different extraction methods. *International Journal of Pharmaceutics*.

[18] Hu J, Chen Y, Ni D (2012). Effect of superfine grinding on quality and antioxidant property of fine green tea powders. *LWT-Food Science and Technology*.

[19] Valerga J, Reta M, Lanari MC (2012). Polyphenol input to the antioxidant activity of yerba mate (Ilex paraguariensis) extracts. *LWT-Food Science and Technology*.

[20] Linares AR, Hase SL, Vergara ML, Resnik SL (2010). Modeling yerba mate aqueous extraction kinetics: influence of temperature. *Journal of Food Engineering*.

[21] Zhou DR, Chen YQ, Ni DJ (2009). Effect of water quality on the nutritional components and antioxidant activity of green tea extracts. *Food Chemistry*.

[22] Bae MS, Lee SC (2010). Effect of deep sea water on the antioxidant activity and catechin content of green tea. *Journal of Medicinal Plant Research*.

[23] Kumazawa S, Taniguchi M, Suzuki Y, Shimura M, Kwon M, Nakayama T (2002). Antioxidant activity of polyphenols in carob pods. *Journal of Agricultural and Food Chemistry*.

[24] Shyu YS, Lin JT, Chang YT, Chiang CJ, Yang DJ (2009). Evaluation of antioxidant ability of ethanolic extract from dill (*Anethum graveolens* L.) flower. *Food Chemistry*.

[25] Zhang Y, Wang ZH (2009). Phenolic composition and antioxidant activities of two Phlomis species: a correlation study. *Comptes Rendus*.

[26] Erkan N, Ayranci G, Ayranci E (2008). Antioxidant activities of rosemary (*Rosmarinus Officinalis* L.) extract, blackseed (*Nigella sativa* L.) essential oil, carnosic acid, rosmarinic acid and sesamol. *Food Chemistry*.

[27] Mossion A, Potin-Gautier M, Delerue S, Le Hécho I, Behra P (2008). Effect of water composition on aluminium, calcium and organic carbon extraction in tea infusions. *Food Chemistry*.

[28] Huang WY, Lin YR, Ho RF, Liu HY, Lin YS (2013). Effects of water solutions on extracting green tea leaves. *The Scientific World Journal*.

